# *LINGO1* Variants in the French-Canadian Population

**DOI:** 10.1371/journal.pone.0016254

**Published:** 2011-01-11

**Authors:** Cynthia V. Bourassa, Jean-Baptiste Rivière, Patrick A. Dion, Geneviève Bernard, Sabrina Diab, Michel Panisset, Sylvain Chouinard, Nicolas Dupré, Hélène Fournier, John Raelson, Majid Belouchi, Guy A. Rouleau

**Affiliations:** 1 The Centre of Excellence in Neuromics, CHUM Research Center and the Department of Medicine, Université de Montréal, Montreal, Québec, Canada; 2 CHUM and the Department of Medicine, Université de Montréal, Montreal, Québec, Canada; 3 Faculty of Medicine, Université Laval, Department of Neurological Sciences, Centre Hospitalier Affilié Universitaire de Québec-Enfant-Jésus, Québec City, Québec, Canada; 4 Genizon BioSciences, St-Laurent, Québec, Canada; 5 Sainte-Justine Hospital, Montreal, Québec, Canada; Ohio State University Medical Center, United States of America

## Abstract

Essential tremor (ET) is a complex genetic disorder for which no causative gene has been found. Recently, a genome-wide association study reported that two variants in the *LINGO1* locus were associated to this disease. The aim of the present study was to test if this specific association could be replicated using a French-Canadian cohort of 259 ET patients and 479 ethnically matched controls. Our genotyping results lead us to conclude that no association exists between the key variant rs9652490 and ET (*P_corr_* = 1.00).

## Introduction

Essential tremor (ET), with an estimated prevalence around 5% in persons aged over 65 years, is the most common movement disorder of adults [1]. Although segregation analysis of ET in families strongly supports the contribution of genetic factors [1], genomewide linkage studies have failed to identify ET-susceptibility genes. This lack of success may be attributable to a complex mode of inheritance involving environmental factors and multiple low-penetrance susceptibility alleles [2]. Recently, a genome-wide association study (GWAS) was conducted by Stefansson *et al.* using 452 ET cases and 14,394 controls from the Icelandic population [3]. They found two single nucleotide polymorphisms (SNP) that were associated with ET (rs9652490 and rs11856808), both in intron 3 of the leucine-rich repeat neuronal 6A (GenBank: BC068558.1 *LINGO1*) gene. The aim of the present study was to attempt to replicate these findings in another population.

## Results

A total of 738 individuals from the province of Québec, Canada were genotyped for rs9652490 and rs11856808. Genotyping success rate was above 98% and both SNPs were in Hardy-Weinberg equilibrium. Minor allele frequencies (MAF) from our control group were consistent with the MAF reported in the GWAS report [3]. Neither genotype counts nor allele frequencies differed between cases and controls (Table 1). An analysis of a subset ET, familial cases, revealed no significant association with the two SNPs.

**Table 1:** Genotype and allelic distribution of rs9652490 and rs11856808 among ET cases (n = 259) and controls (n = 479).

SNP	HWE	Genotype/Allele	Patients N (%)	Controls N (%)	*P-values ([Table-fn nt102] 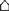 Corrected)
**rs9652490**		A/A	153(59)	287(61)	
		A/G	94(37)	160(34)	
		G/G	10(4)	23(5)	0.91 (1.00)
	0.75	MAF All (G)	0.22	0.22	0.95 (1.00)
	1.00	MAF FET (G)	0.23	0.22	0.71 (1.00)
**rs11856808**		C/C	117(46)	204(43)	
		C/T	105(42)	204(43)	
		T/T	29(12)	63(14)	0.34 (0.68)
	0.24	MAF All (T)	0.32	0.35	0.35 (0.70)
	0.23	MAF FET (T)	0.34	0.35	0.81 (1.00)

*Cochrane-Armitage trend test for genotype comparisons and Fisher's exact test for allele comparisons.

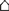
Correction for multiple testing using Bonferroni's correction.

HWE. Hardy-Weinberg equilibrium exact test.

MAF. Minor allele frequency.

FET. Familial Essential Tremor.

## Discussion

Our association study using a French-Canadian cohort of ET cases does not support an association between rs9652490 or rs11856808 and the disease. Moreover this lack of association is also true when only the subset of patients with a familial history of ET is examined, contrarily to other studies discussed below. It is noteworthy that the size of our cohort is comparable to the size of cohorts used by other groups who also tested if the association with rs9652490 could be replicated.

Our power to detect an association of low impact is limited because of the sample size available. Therefore, the lowest odds ratio (OR) we could ascertain from it is 1.54. Other replication studies published had lower ORs (1.33, 1.28) but when looking at their results closely, the 95% confidence interval included 1.00. Because this means there is no relationship between the parameter tested and the disease, we chose not to base our power calculations on these ORs (see Materials and Methods section).

To have an 80% power to detect an effect of the rs9652490 variant in the French-Canadian population with the current relative risk observed, it would require around 40,000 cases with the same control to case ratio [4]. This is highly indicative of how low the effect of the variant is on the genetics of ET in the French-Canadian population.

The few studies that have also attempted to replicate the results of the original report are the following [3], [5], [6], [7], [8], [9], [10]: One where ET cases from both Germany and France were used where a significant association was found between the G allele of rs9652490 and ET in the German cohort; a result they replicated using the French cohort (*P = *0.009 OR = 1.61 [1.21–2.14]) [5]. The same study also conducted a separate analysis of familial ET cases versus the controls and observed a stronger signal. A second study by Tan *et al.* investigated case-control series of ET from Singapore and a significant association between the G allele and the phenotype was found only in a subset of patients with familial history of ET (*P* = 0.007 OR = 1.69); when using the entire cohort they found a non-significant but suggestive association between G allele of rs9652490 and ET (*P* = 0.068) [6]. In a third study conducted using a non-hispanic North-American cohort of ET patients the association between ET and rs9652490 was also marginal (*P* = 0.0569 OR = 1.33 [0.99–1.80]) [7]. Altogether these three studies supported, to a certain extent, the trend observed in the original paper suggesting that the G allele was associated with ET.

Conversely two different studies investigated the association with rs9652490 using North-American ET and PD patients [8], [9]. The ET cohort investigated in both studies consisted of 349 and 353 patients, respectively. The two studies were conducted by the same group, therefore we will discuss the results of the first report only: the A allele of rs9652490 was the risk allele for both disorders (*P = *0.0145 OR = 2.2), compared to the G allele reported. It is unlikely that these conflicting results are due to population-specific susceptibility variants as the follow-up group used in the original report to validate the association between the G allele of rs9652490 and ET included individuals from the United States and Europe [3]. This variability might by due to the “flip-flop” phenomenon, which occurs, in complex traits, when the associated allele is in weak linkage disequilibrium (LD) with the true causal variant. Even if the samples are from the same origin, environmental factors might contribute to the association with the other allele [11].

The last study was conducted using Chinese ET and PD cohorts and no association was found between neither ET, or PD, and *LINGO1* (*P* = 0.910 OR = 1.022 [0.706–1.477]) [10]. However, this negative study probably does not result from population differences, as Tan *et al*. had failed to observe any differences in linkage disequilibrium of markers around rs9652490 between Asians and Caucasians [6].

Even if we know that the French-Canadian population is quite homogeneous, we did not test LD between the North-American population (from the United States and Canada), the French population and French-Canadian one. Although we cannot exclude a difference in LD between the French-Canadian population and other ones, if rs9652490 is a marker in LD of the true causal variant, which is the hypothesis prevailing in this case, [5], [7], [8] we would have seen an association with either the G allele or the A allele, as reported by other groups. Moreover, for complex traits a possible “synthetic association” may be seen. The concept of such an association relies on the association seen between a common variant and rare causal ones [12]. In synthetic association, it was shown that true causal variants were not necessarily in the same LD block as the common associated SNP. Therefore, even if we did not test LD in *LINGO1* in the French-Canadian population, a true association could have been revealed if there was a suggested one.

The aim of this report was solely to replicate the findings of the first GWAS describing an association in ET patients, using French-Canadian ET patients, and so no direct sequencing or genotyping of additional SNPs was done for the whole *LINGO1* gene. Such broader experiments could be done in a near future to see if another association between *LINGO1* and ET could be identified in the French-Canadian population.

Meta-analyses were recently reported by Tan *et al.*, Clark *et al.* and Vilariño *et al.* and so even if our results are revealing about lack of association in the French-Canadian population, a new meta-analysis with only these new French-Canadian cases would not be significantly different yet [6], [7], [8]; more replication studies would be needed before such a meta-analysis is needed.

In conclusion, our data, which does not support an association between rs9652490 or rs11856808 and ET in French-Canadians, and parallel replication studies, argue against a definitive role for these *LINGO1* polymorphisms in ET. Furthermore it is important to keep in mind that even if rs9652490 showed the highest association signal in the original report, none of the markers genuinely reached a genomewide significance level in this study (*P = *3.0×10^−7^ and 1.6×10^−7^ was expected) [3]. A GWAS using a much larger sample size would be needed to assess whether common genetic variants predispose to ET.

## Materials and Methods

### Ethics Statement

The local institutional committee, Comités d'évaluation scientifique et d'éthique de la recherche of Centre hospitalier de l'Université de Montréal (CHUM) approved of this project: Identification of the Gene Predisposing to Tremor, following the Declaration of Helsinki. Informed written consent was obtained from each participant.

### Subjects

A total of 259 patients with ET (104 with familial history of ET) and 479 ethnically-matched population controls were included in this study. All individuals were recruited in the province of Quebec, Canada, and probands were ascertained by neurologists specialized in movement disorders. Informed written consent was obtained from each participant and the study was approved by the local institutional ethic committees.

### Genotyping

Markers rs9652490 and rs11856808 were genotyped by TaqMan SNP Genotyping Assay following the manufacturer's instructions and results were assessed using the Applied Biosystems 7900 Fast Real-Time PCR System and SDS software (vs. 2.2.2) for allele calling.

### Statistical analyses

The Hardy-Weinberg equilibrium exact test was performed to ascertain the normal heterogeneity of the population for the two markers tested. Case-control association study was performed using the Cochran-Armitage and the Fisher's exact tests. Correction for multiple comparisons testing was made using Bonferroni's algorithm as the two SNPs tested were in LD. All these tests were done using PLINK software [13] (vs.1.07). Power to detect association was determined using the Genetic Power Calculator and, if the effect of the variant was as strong as the effect seen in the Icelandic population (OR of the G allele: 1.63), this cohort would be of sufficient size to get an 88% power of detection [4].
